# Occupational Therapy Home Safety Intervention via Telehealth

**DOI:** 10.5195/ijt.2016.6183

**Published:** 2016-07-01

**Authors:** LORI E. BREEDEN

**Affiliations:** SCHOOL OF OCCUPATIONAL THERAPY, COLLEGE OF HEALTH SCIENCES, UNIVERSITY OF INDIANAPOLIS, INDIANAPOLIS, INDIANA, USA

**Keywords:** Home safety, Older adults, Photo-elicitation, Telehealth

## Abstract

Photography can be an effective addition for education-based telehealth services delivered by an occupational therapist. In this study, photography was used as antecedent to telehealth sessions delivered by an occupational therapist focused on narrative learning about home safety. After taking photographs of past home safety challenges, six participants experienced three web-based occupational therapy sessions. Sessions were recorded and transcribed. Data were examined using content analysis. The content analysis identified the following themes: the value of photos to support learning; the value of narrative learning related to home safety education; and abstract versus concrete learners. Procedural findings are included to support future endeavors. Findings indicate that within a wellness context, home safety education for older adults can be delivered effectively via telehealth when using photography as a part of an occupational therapy intervention.

According to the workforce survey by the American Occupational Therapy Association (AOTA, 2010), most occupational therapists, approximately 55.4%, work in a direct style of service delivery wherein a therapist provides one-to-one treatment in a medical setting. Given the current trend in health care toward shorter hospitalizations, and recent reform legislation, occupational therapists are exploring different models of service delivery. In telehealth, a new type of service delivery for many occupational therapists, the methods and quality of client education can be important factors in determining successful client outcomes.

Patient education in a clinical setting is often guided by an occupational therapist’s understanding of activity analysis, use of adaptation, and the structuring of tasks that provide the best challenge for patients (Dahlin-Ivanoff, Sonn, & Svensson, 2002; Schemm & Gitlin, 1998). These methods provide a client with opportunities to learn through practice with a skilled clinician present. Telehealth practice can challenge occupational therapists to use different methods of client education that do not rely on hands-on assistance or practice. There is a lack of evidence to support educational strategies in telehealth practice. To address this gap, this multi-case study examines the experience of educating older adults about home safety via telehealth using photography as an antecedent to narrative learning.

The central research questions were as follows:

- What is the process of home safety education with older adults as delivered via telehealth?
- Does the combination of digital photographs and narrative learning impact home safety as scored by the Safety Assessment of Function and the Environment for Rehabilitation-Health Outcome Measurement and Evaluation, version 3 (SAFER-HOME v. 3)?
- How does the use of digital photography facilitate narrative learning and impact individuals’ awareness of safety challenges in their home?

One of the many challenges in occupational therapy is a lack of adherence by older adults with regard to home safety. This is especially challenging when working with community dwelling, independent older adults whose range of performance is decreasing but who are otherwise functioning well. They may not see a reason to change their environment or habits when they have not yet experienced difficulties with safety. These clients can be very resistant to recommendations from health care practitioners about safety in their homes. Helping older adults anticipate their changing function by balancing knowledge of past functioning with a realistic view of their future may increase their openness to suggestions for home safety.

Involving these clients in decision-making processes can facilitate adherence. Clemson, Cusick, and Fozzard (1999) stated, “the ownership of ideas and exerting control within the context of an individual’s environment and life experiences strongly influences acceptance and follow through of recommendations” (p. 539). Additionally, these researchers recommend “joint decision making and negotiation” (Clemson et al., 1999, p. 539) as being most likely to result in changed home environments. Radomski (2011) recommends that adherence is a function of the client, the provider and the recommendations set within the environment; and that adherence is a process of client and provider decisions rather than recommendations.

## FALL PREVENTION

Of great concern when working with community dwelling older adults are the physical and psychological injuries that accompany a fall. The Centers for Disease Control and Prevention (CDC) (2010) reported that $19 billion was spent in 2000 on treating the elderly for the adverse effects of falls. It is estimated that this equates to $30 billion in 2010 dollars (CDC, 2010). Relevant to occupational therapy, injury from a fall can slow occupational performance at home as well as on the worksite and can contribute to other fall-related costs to individuals, families, and organizations such as lost time on the job, inability to perform household activities, and decreased quality of life (CDC, 2010). Fleming (2002), identified falls as the leading cause of death among people 65 years and older. In the year 2000, non-fatal falls accounted for 19 billion dollars in health care expenses (Stevens, 2006)

The definition of a fall varies by institution; however, a common element seems to be unintentionally coming to the ground, floor, or other lower level (Tinetti, Baker, Dutcher, Vincent, & Rozett, 1997). Effective interventions to prevent future falls assume falls are not the result of random accidents but rather result from the presence of numerous risk factors and a compromised medical condition (Tinetti et al., 1997). It is therefore essential to help community dwelling older adults examine their environments and improve home safety. One clinical strategy that therapists use when working with a client who has fallen is to ask the client to recreate the fall. This process is often used to help a therapist appraise past performance; an additional benefit is that the client uses narrative learning to examine his or her safety.

## NARRATIVE LEARNING

The use of a personal narrative is a way people reason, and make meaning out of an event. Through the process of telling the stories of their lives people reason about cause and effect. Clark (2010) stated, “we learn from telling stories. When we hear, we are the receiver; when we tell, we are the actor, the one putting all the details together and making the experience coherent for ourselves and for others” (p. 6). Learning via narrative is an integral part of adult learning theory. Lindeman (1989) offered, “Adult education is a process through which learners become aware of significant experience. Recognition of significance leads to evaluation. Meanings accompany experience when we know what is happening and what importance the event includes for our personalities” (p. 109). For a client recovering from illness or injury, the process of organizing thoughts and crafting the story of one’s experience helps to evaluate the process and assign meaning.

Clark and Rossiter (2008) position narrative learning within constructivist learning theory, which “understands learning as construction of meaning from experience. The fundamental principles of narrative underlie this type of learning because the meaning construction is done narratively” (p. 63). Narrative learning occurs because of the processes that individuals must use to organize their thinking such as highlighting the relevant points of the experience and building those relevant points into a narrative. This process helps the person to both solidify the experience and to mentally construct a perspective of the event that creates its meaning. As we listen to an individual telling his or her story, we witness learning in real time as the narrator relates the experience. Personal narratives are a way to structure and make meaning of daily lives and can facilitate a transformative and critically reflective understanding of those experiences (Clark, 2010).

Schank and Berman (2006) note how much of the educational process is comprised of telling stories, even though most listeners do not retain what they hear:

It is rare that we learn from others’ stories, but stories play a key role in our day to day conversation. Why do we tell stories if others will not remember them? The answer is that we like to hear ourselves talk, and actually, we learn from hearing ourselves talk. So we (pretend to) listen to others to get them to listen to us. We are not consciously pretending, but we also are not really gaining much from what we hear. (p. 220)

Applying this notion, telling clients how to be safe at home may not be as effective as having clients tell clinicians how breaches in home safety occurred. When clients listen to suggestions and advice regarding home safety, that advice may not be heard; yet when clients themselves explain, even a flawed process, they likely derive benefit.

## PHOTOGRAPHY

In this study participant-generated digital photographs were employed as an antecedent to participants’ narrative learning about home safety. The photographs, used as an archival record, allowed participants not only to share a visual image representing the past safety challenge but also to provide a basis for the description of their experience. Harper (2002) defined photo elicitation as “the simple idea of inserting a photograph into a research interview” (p 13). Collier and Collier (1986) described taking notes during a traditional interview as something that would “block conversation” (p. 106). Additionally, the authors described the use of a tape recorder as something that would “bring all conversation to a stop” (p. 106). Collier and Collier (1986) were supportive of the use of photographs; using them seemed to focus all discussion toward the photograph. Furthermore, neither note taking nor recording seemed to bother participants when conversation was centered on a photo instead of the participant. As Collier and Collier (1986) explained:

Photographs sharpen the memory and give the interview an immediate character of realistic reconstruction. The informant is back on his fishing vessel, working out in the woods or carrying through on a skilled craft. The projective opportunity of the photographs offers a gratifying sense of self-expression as the informant is able to explain and identify content and educate the interviewer with his wisdom. (p. 106)

One reason that photo elicitation could support both reflective and critical reasoning skills is that examining a photo allows for a perspective on a point in time from which the photographer is removed. Allowing the participant to reflect on a situation from a more objective, even safe point in time can support critical reflection of past accidents.

Latz (2012) discussed the unintended results of a study using photovoice to elicit critical consciousness in community college students. The author illustrated how participant-generated photographs in research elicited not only critical consciousness but reflective consciousness as well. Latz (2012) noted: “Photovoice for reflective consciousness may be used as a tool for inquiry as well as personal development” (p. 50). This article is relevant to the current study in that participants were asked to use reflective consciousness, elicited by the photographs, to develop their understanding of home and community safety.

As occupational therapists employ telehealth, it is important to understand the educational tools that are available and how the educational process can influence a client’s outcome. In this study, photography and the accompanying client narrative construct a learning experience about home safety. Using photographs to promote narratives could help the occupational therapists guide home safety education toward specific issues that the older adult has deemed relevant.

## METHODS

Subjectivism respects that older adults have an intimate understanding of their homes and how they function. Crotty (1998) noted, “In subjectivism meaning does not come out of an interplay between subject and object but is imposed on the object by the subject” (p. 9). This foundation encouraged an examination of home safety as clients interpret the issues through their own experiences. Drawing on strengths from both qualitative and quantitative research, a “multicase study” (Stake, 2006, p. vi) design with a primarily qualitative focus was used. Narrative learning data was supplemented by the use of a pre and post measure of home safety, the Safety Assessment for Function and the Environment for Rehabilitation – Health Outcome Measurement and Evaluation (SAFER HOME) v. 3, (Chiu et al., 2006).

### PARTICIPANTS

Participants were six “purposefully selected” (Creswell, 2013, pp. 299–300), older adults over the age of 65 who were recruited via email or a recruiting flyer distributed through personal and professional contacts. Inclusion criteria included a fundamental knowledge of digital or cell phone photography, computing, and email use. It was important that participants were comfortable with technology, to be able to improve their understanding of home safety while at the same time managing the technology. Individuals who had experienced recent health problems were excluded if they had previous exposure to occupational therapy services focused on home safety. After emailing their interest in participating in the study, six older adults were contacted by phone. Each agreed to participate in the study and were scheduled for an initial meeting. All participants were retained for the duration of the study.

### MATERIALS

The SAFER-HOME v. 3 (Chiu et al., 2006) was used pre- and post-intervention. This is a 74-item assessment that scores individuals as having no, mild, moderate or severe safety concerns based on interview or observation of household tasks. The SAFER-HOME v. 2 is reported to have “high internal consistency with a coefficient alpha value of 0.859, indicating that the 97 items all contributed to the measurement of one dimension (home safety)” (Chiu & Oliver, 2006, p. 140). SAFER-HOME v.3 is the current version and has fewer items, less categories and a change in weighting of categories to better capture more severe problems. These changes, based on expert reviewer feedback have made the tool more responsive to change over version 2 (Chiu, et al., 2006).

Three weekly telehealth sessions were performed using the VSee software program (VSee, 2013). VSee is a web-based video technology currently recommended for telemedicine because of its ease of use and security. Tsuboi noted, “As far as privacy concerns, VSee uses end-to-end encryption.” (2013, para. 4). This process ensures that data being transmitted is not decrypted on a remote server, and is therefore only accessible by the end users. VSee requires that participants have access to Windows XP SP3, Windows Vista or later versions, Mac OSX 10.6 and above, or iPad iOS 5.0 or later.

### PROCEDURES

Following recruitment, an initial in-person session was scheduled in the participant’s home. During the initial session, informed consent was obtained and the researcher performed baseline assessment using the SAFER-HOME v. 3. Participants were then instructed to create photographs of home safety concerns that would be used to guide future web-based sessions. These were sent to the researcher prior to each telehealth session through email or via text messaging, which allowed for session preparation focused (when possible) on specific issues within the photo. Member checking was used to establish trustworthiness (Creswell, 2013) along with a researcher reflexivity journal, which was used to expose bias and analyze the learning experience from the perspective of the researcher (Lincoln & Guba, 1985).

Following baseline assessment, three sessions were scheduled based on participant availability and administered remotely via web-based video-conference, with the participants communicating from the computer in their homes. During these web-based video sessions, the participants engaged in telehealth sessions based on open ended questions designed from James Spradley’s developmental research sequence (Spradley, 1979), moving from broad descriptive questions through structural questions, and finally into contrasting questions. Sessions included a discussion of how the images in the photographs related to past accidents, near misses or home safety issues. A near miss was defined as “an unplanned event that did not result in injury, illness, or damage – but had the potential to do so” (National Safety Council, 2013). After each telehealth session, a new photo assignment was given, based on the discussion(s) as they evolved in the prior session.

Sessions were audio/video recorded using VSee and transcribed using Microsoft Word^®^ within 48 hours. Participants were assigned an alias during transcription and the session recording was deleted. Following the three telehealth sessions, a final in-person session was scheduled during which the SAFER-HOME, v. 3 was re-administered to quantify any functional or environmental changes that had been made. This process took place over a seven-week period.

### DATA ANALYSIS

Telehealth session analysis included ‘within case analysis’ (Creswell, 2013, p. 101), as well as a ‘cross case synthesis’ (Yin, 2009, p. 156). During an initial round of open-coding, the researcher read transcripts, examining patterns that participants discussed in their photo-supported narratives (Creswell, 2013). This ‘within case analysis’ (Creswell, 2013) was performed immediately following telehealth sessions, and contributed to successive session topic assignments while also informing thematic categories. Content analysis was performed, across cases, on all session transcripts; data were organized categorically, coded and combined with concepts emerging from photographs and researcher reflections. Memos were assigned to provide context to codes. Themes that emerged from the data related to narrative learning and the use of photography were technology use, concrete versus abstract learners, and developing rapport.

The qualitative analysis was corroborated by quantitative data from the SAFER-HOME v.3. This measure was non-experimental and used descriptive statistics to measure pre/post scores related to home safety. Data reflected the participants’ environment(s) and ability to function safely during activities of daily living. The SAFER-HOME v. 3 was administered to obtain information about their individual change over time. These assessment scores were not generalizable but offered an indication that the home safety education program led to individual improvement. Paper copies of de-identified data were scanned and saved electronically, along with photos, transcriptions, and reflexivity journals on a password protected, encrypted file as an audit trail of the analysis.

## RESULTS

Procedural findings reported here are related to the process of providing this very specific home safety program via telehealth, to allow others who attempt client education via telehealth to better prepare for the unique challenges. Examples of participant narratives are offered as examples related to the previously detailed research questions.

### PROCEDURAL FINDINGS

The participant-created photographs, which arrived via email or text prior to the telehealth sessions, were sometimes intentionally staged and very creative, while others were more concrete and focused on current environmental conditions. Likewise, the descriptions provided by the participants about the photos varied based on many factors. These factors included participants’ level of trust and confidence in the researcher, their expectations of the role of the occupational therapist in the process, and their willingness to share stories that exposed past failings. Participants who were comfortable with abstract concepts seemed more willing to engage in reflection; those who seemed more concrete focused on finding a right or wrong answer to their experiences. In this telehealth educational experience, technology was ever present in the learning process, and offered both barriers and supports to learning. Change in the SAFER-HOME, v. 3 scores, and the richness of the participant narratives (discussed below) indicate a benefit from this learning experience.

#### TECHNOLOGY

**Figure 1:**
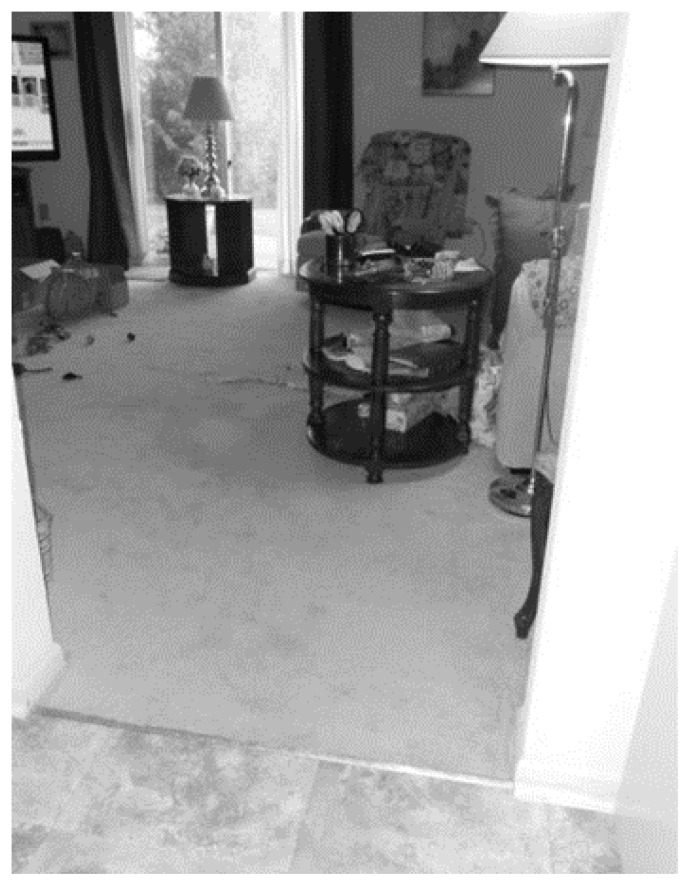
The path of Participant 2’s near fall.

Technology can create a challenge for intermittent users due to constant evolution and inconsistencies in software, which can slow the work process. The older adults in this study were confident and quite comfortable with the use of technology. Yet one challenge technology posed was that occasionally, web-connections were lost, temporarily interrupting a telehealth session, as in the following example. In a telehealth session focused on a stumbling fall, augmented with the photo in Figure 1, Participant 2 explained:

It was just like I was diving forward and I kept flailing with my arms and kept going and kept going and I thought oh my God I’m going to go straight through the window. But I managed to get stopped because there is a chair there and a good heavy table and I managed to get stopped without going down on even one knee and uh—I was just trying to get my balance and (disconnect). (Session one)

Once we reconnected, I prompted Participant 2 to finish and she offered, “well yeah, well I don’t know what else— that is really all there is about that.

In a study focused on learning through the narration of a story, losing connection mid-story was disruptive and participants were less detailed once the connection resumed. Overall, the older adult participants did not seem to allow technology challenges to interfere with the session and often managed to laugh at themselves without internalizing any of the challenges.

#### SELF-DISCLOSURE

Building a trusting and comfortable relationship to put clients at ease and foster self-disclosure occurred in an intentional manner in the online format. It was important for participants to feel confident that the role of the researcher was not to impose change, nor report safety challenges to a participant’s family. The degree of self-disclosure differed across clients.

**Figure 2:**
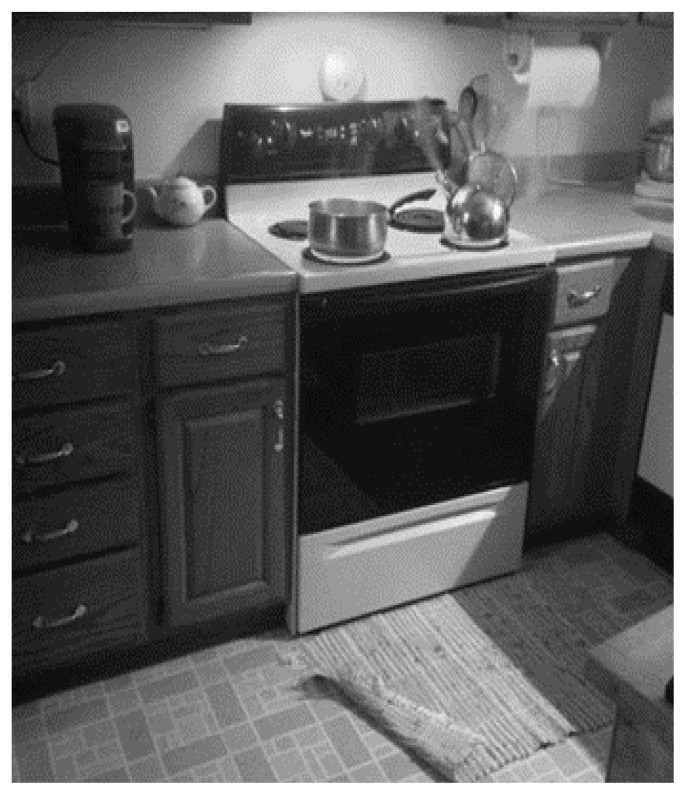
Participant 1 photo represented numerous times that she forgot items cooking. In the third telehealth session (session four overall), this participant newly disclosed the details of a cooking accident, insisting those events were rare or even one-time occurrences.

Participant 1 was politely participatory during sessions. However, it was not until the third telehealth session that this participant seemed to embrace the process of selecting and staging a photo containing several salient elements. During the concluding session, Participant 1 finally seemed comfortable sharing her mistakes and offered the following narrative, prompted by the Figure 2 photo:

You know what I did this weekend—just three days ago? I was taking hot food over to my daughter’s, we were serving a Chinese meal and we wanted everything good fresh and hot, so I was putting everything in my cooler, and I had my hot food in there and I put a rice bag in the microwave for three minutes because I wanted it good and hot and I was putting my coat on and I thought, what is burning, the stove isn’t even on and a couple minutes later I thought, something is burning? And then I remembered the microwave! And the rice bag was burned, the cloth actually burned. See there was some time left on the microwave when I put it in, so it could have gone for 30 minutes—I don’t know— I could have burned the house down! (Session three)

Participant 1 also related another accident scenario in the third telehealth session. As a workman entered the home, he slipped off the front landing and fell into the dining room table, hitting his head. These events further encouraged her interest during the final in-person visit. Participant 1 engaged in nearly three hours of home safety discussion, primed by recent events. A level of trust with this participant had taken some time to establish, yet she ultimately experienced transformative learning because of the telehealth presence of a clinician in her home.

A positive example of building a relationship that fostered self-disclosure was found with married Participants 3 and 4. This couple participated together and seemed to share the learning, planning, and the decision-making related to home safety. Their rich narratives were therefore therapeutically productive.

While Participant 5 was friendly, she was more reticent to self-disclose content. She discussed topics she was familiar with–but admittedly would likely do nothing about. She photographed throw rugs, and indicated that she knew they were a trip hazard, but she was keeping them. This resistance to change included her beer drinking, which she indicated she had no intention of changing. Participant 5 was very adept in steering the direction of a conversation, and deflected questions that asked her to reason about her drinking. She used humor to frame a discussion on driving in Atlanta traffic to visit family. However, as the story unfolded it became clear that when and where she would be able to get off the road and enjoy “some suds” was an underlying part of the travel plan:

We had discussed going a ways and stopping and spending the night, but it’s like I said, who wants to stop at two o’clock in the afternoon. Unless we could just leave home later in the day and get in there about five o’clock, the traffic through Chattanooga is not bad. Even Nashville doesn’t bother me so we could get in there kind of late, five or six o’clock in the afternoon and then walk around. ‘Cause when I get out of the car I like to walk around anyway, stretch my legs and then, have myself go back, and have a couple of beers, and then go eat supper. Well by then it’s eight or nine o’clock and I’m ready to relax. (Session three)

While it is possible that Participant 5’s effort to control the flow of discussion was related to the quality of the rapport, it is possible that she was trying to avoid the transformative experience that narrative learning, through reflection, could achieve.

### OUTCOME MEASURE

Improvements in SAFER-HOME v. 3 scores, although slight, occurred for five of six participants in this study. Because of the small sample, it is not possible to draw specific cause and effect relationships between the photo-elicited narratives and changes in the overall SAFERHOME, v. 3 Assessment. While the SAFER-HOME, v. 3 remains a good choice for this type of comparison, this research project is exploratory in nature and the data indicating that this process contributes to a safer environment is found only in descriptive subsections of this assessment tool. In some cases, SAFER scores on specific line items did not improve after greater understanding of how an individual participant functioned within their space. This occurred with Participant 1 during the initial session when it was noted that the bathtub had a non-skid surface and grab bar. A discussion on her bathing preferences and a past fall, combined with her resistance to consider changes, indicated she was less safe in that category, resulting in a poorer score.

This assessment tool determined whether individuals were making use of their learning in a functional way. Most participants, as indicated in Table 1 below, showed slight improvements often attributed to reducing clutter that allowed for a wider pathway or reducing trip hazards.

#### PHOTOGRAPH USE

The process of photo elicited narrative learning made an impact on these participants in that it focused their reflection on past safety experiences. The use of the photos and the stories that the participants shared seem to personalize the topic and provide a foundation for future planning. Rather than looking at a pamphlet with instructions on how to make a home safer, photos created by the participant made the experience more applicable to them and, therefore, a more “authentic learning” experience (Ravitch, 2010, p. 23). Past experiences captured in the photo meant that all of the items addressed were issues that were relevant because they were events from the participants’ lives. However, the concept of digital photography in support of narrative learning as a way to focus reflection was a different experience for each participant. A greater depth of discussion seemed to accompany a more abstract or sometimes staged photo, and a more concrete photo seemed to be associated with participants who simply wanted recommendations on a topic that had challenged them in the past.

#### ABSTRACT VERSUS CONCRETE PHOTOS

Participants needed an example to focus their photos on a specific topic or area, such as kitchen safety. For some participants this additional prompting was not adequate, and they needed specific examples. For example, if a participant wanted to discuss a time when they fell, the researcher suggested they should try to take a picture of the area, or set up something in the photo that would remind them about the experience.

**Figure 3:**
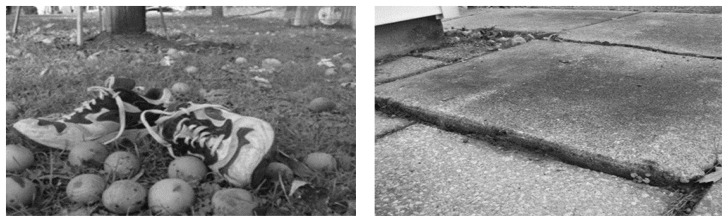
The more abstract photo on the left was staged recalling a time when stepping on a fallen walnut led to a twisted ankle. On the right is a more concrete photo, showing an uneven concrete surface as a trip hazard.

Examples of abstract and concrete photographs are found in Figure 3. Participants 3 and 4 were the most enthusiastic and seemed to embrace the abstract use of a photograph. Other participants, at some point, were challenged to know what to include and sought out the researcher’s ideas. Participant 6, stated during the second session: “I don’t know what we will talk about—I’m really just a couch potato” and “Well I looked over the house and I couldn’t find anything I thought that was a real problem, except what I call, those little choke points that you might have a problem getting through.”

Participant 5 struggled with the abstract nature of the photo assignment:

Participant 5: A picture of something—well, that’s going to be—I’ve had a driving challenge before and, in several ways.

Researcher: Okay.

Participant 5: I’m not sure, I’m not sure I can send you a picture of like ice on the road.

Researcher: Okay.

Participant 5: I don’t know how I’m going to send you a picture of that.

Researcher: Well, you don’t have to, just something that reminds us, something to get us talking.

Participant 5: I could send you a picture of my car and that would get us talking.

Researcher: Okay. Whatever inspires you.

Participant 5: Would that be okay?

Researcher: That would be fine. It’s up to you. (Session two)

**Figure 4:**
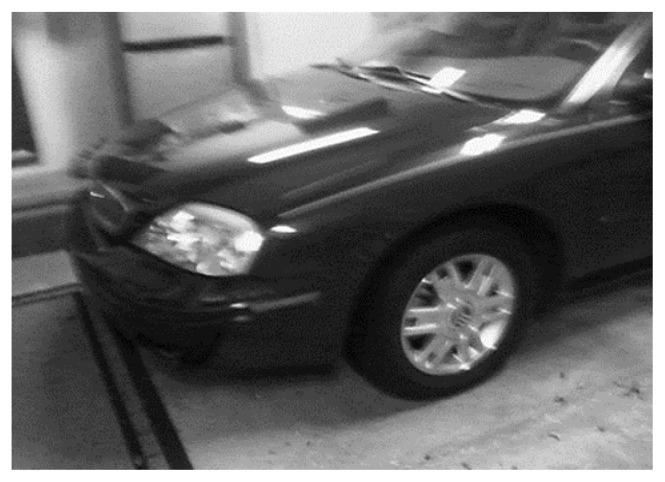
Participant 5’s car parked in the garage.

This exchange led to a very concrete photo of Participant 5’s car parked in her garage seen below in Figure 4.

Participant 5 seemed to maintain a focus throughout that she was part of the study to help out the researcher, and would do whatever was needed. This helpful perspective seemed to limit her ability to reflect and learn from her experiences.

Participant 6 seemed to see his role as the family’s problem solver. Each time he and his wife experienced a safety problem, he sought out advice and implemented a change. This process worked well for Participant 6 and his wife, so he was less inclined to dive deeper into a reflective experience and tie the past safety issues to potential future issues. He perceived the problem to be fixed after changes were made.

## DISCUSSION

Current findings were examined in light of existing literature on older adults and the use of technology, and building rapport; using photography as part of narrative learning with abstract versus concrete learners; and collaborative cognition. Study limitations as well as recommendations for occupational therapy practice via telehealth follow.

### TECHNOLOGY

Findings in this study indicate that older adults were quite comfortable with the use of technology. Broady, Chan, and Caputi (2010) examined existing literature related to attitudes and abilities with computers and essentially found that the attitude and experiences of young and old are similar, countering the popular myths about older adults and computer use. The authors identified several factors that are likely to enhance an older person’s experience with computers. These include allowing ample time for skill mastery, treating the older adult user/learner in a positive manner that makes them feel valued, and the teacher’s expectation of success.

Werner, Carlson, Jordan-Marsh, and Clark (2011) examined some of the psychosocial characteristics and coping skills of older adults who were and were not active computer users. These researchers stated:

Older adults who tend to be actively involved, rather than withdrawn, were more likely to use computers. Correspondingly, active coping approached significance as an independent predictor of greater general computer use. These results may be indicative of a dispositional proactive approach to challenges, such as learning to use technology later in life. (pp. 442–443)

Werner et al.’s work (2011) is relevant to this study as the older adults who reported they were comfortable with technology were also active and engaged with their families and other social circles, and participated in some form of work or study on a regular basis.

### RAPPORT

The experience of this study suggests that the building of rapport in a telehealth setting to facilitate participant self-disclosure, requires intentional effort and skill. Rogers defines rapport as “positive, synchronous interactions that reflect closeness or connectedness in a relationship” (2015, p. 20). Tickle-Dengan and Rosenthal (1990) identified nonverbal behaviors as a key element in creating rapport. For instance, they identified “bodily postures” (p. 290) that indicated attentiveness and positivity such as smiling and head nodding to be nonverbal behaviors that support rapport. In this study, these mannerisms, required the therapist/researcher to focus their gaze on the web cam, rather than the image on the computer monitor. It was easy to drift away from this view and look at the face of the participant. This action, although repeatedly corrected, perhaps gave the participants the feeling that the therapist/researcher was gazing down and not looking directly at them.

While rehabilitation therapists use therapeutic touch to build rapport, a study by Morris Henegar, Khanin, Oberle and Thacker (2014) indicates that touch is not always perceived as intentional. These researchers examined the use of touch by occupational therapy practitioners and found that 80% of all therapeutic touches given were “instrumental in nature,” and used with handling techniques rather than the “expressive touch” which is more likely to build rapport and client self-esteem (2014, p. 140). Understanding that human touch is a method of developing rapport, one must recognize that it is often not intentionally developed within in-person sessions in a therapeutic manner. It is important to build rapport in both telehealth and in-person service delivery. The use of video recording and post session reflection offers the practitioner an opportunity to improve rapport-building skills in telehealth practice.

### PHOTOGRAPHS

No evidence was found in the literature of photo-elicited narrative learning directly influencing home safety. However, Gidman (2013) wrote about the use of patient storytelling to help student nurses understand their patients’ experiences. The author recommended that while student nurses learn from the storytelling experience, it is up to educators to understand this educational process and build upon its value by facilitating student reflection. The findings, as well as the improved SAFER-HOME, v. 3 scores, support the value of combining photographs and narrative. The reflection experienced while taking photos and then discussing the stories behind the images was powerful. This reflection supported changes in insight as well as environment.

#### CONCRETE VS ABSTRACT

The example of a concrete photographs (Figures 3 and 4) were considered through the lens of a study by Janzen, Perry, and Edwards (2011) who stated, “Photovoice as an artistic pedagogical technology supports the existence of authentic voice, fulfills the criteria of being an authentic medium, and provides a channel for authentic statements” (p. 12). These researchers question the possibility of creating a “social presence” by projecting oneself in an online educational experience. This social presence is important in developing cohesive collaborative communities that facilitate unguarded communication (Garrison, 2007). Participants 5 and 6’s photographs, quite concrete in nature, did not open a door to relationship building and therefore did not add a social presence to this educational experience— nor did they help facilitate an unguarded conversation. Participant 6 took a photo because he was asked to, and simply offered a “show and tell” type story with no anticipation that he may derive future benefit from the experience.

Naufel and Beike (2009) examined abstract and concrete thought in terms of goal performance. They found that when individuals created concrete goals they focused on how the goals were implemented; individuals who created abstract goals were focused on the reasons why the goals were implemented. Certainly the environmental nature of home safety can be a concrete issue, and some participants struggled to find an abstract way to explore this topic. Creativity seemed to emerge when there was genuine curiosity about the subject.

#### COLLABORATIVE COGNITION

Participants 3 and 4 had been married for many years and shared the telehealth experience. They provided a unique view into what previous researchers described as collaborative cognition (Berg, Johnson, Meegan, & Strough, 2003; Dixon, 1996; Dixon & Gould, 1996). Strough, Cheng, and Swenson (2002), regarding collaborative cognition, stated, “solving problems with other people may allow older adults to compensate for age-related declines in their problem-solving ability and age successfully” (p. 26).

Within the telehealth sessions, Participants 3 and 4 discussed a variety of experiences, solutions, and even friends’ experiences. They addressed more topics with greater depth, than other participants. Mezirow (2000) stated, “transformative learning is a way of problem solving by defining a problem or redefining or reframing the problem” (p. 19). He further stated, “we often become critically reflective of our assumptions or those of others and arrive at a transformative insight, but we need to justify our new perspective through discourse” (Mezirow, 2000, p. 19).

The photo, the recollection of a past event, or possibly a focus on current environmental conditions, helps direct our attention to risk. Turning this attention into a narrative that a person owns and shares is supported by a constructivist approach to learning; the creator of the story becomes the expert. This process is inherently different from the didactic approach to educating patients that is common in health care, which encourages the patient to be the passive receiver of information not an expert in creating a safe space. In occupational therapy the paradigm expressed in Mary Reilly’s statement “Man through the use of his hands, as they are energized by mind and will, can influence the state of his own health” (Reilly, 1962, p. 2) continues to hold meaning in this study. It was not through clinical advice that the participants came to understand safety challenges in their home, but through knowledge created with their photographs and narratives.

## CONCLUSION

The purpose of this study was to explore the experience of a home safety education program for older adults, grounded in narrative learning and delivered electronically using digital photographs via telehealth. This study also contributes to the knowledge of how occupational therapists provide client education through telehealth.

### SUGGESTIONS FOR FUTURE RESEARCH

This study has the potential to spur other types of study on the topic of photo elicited narrative learning. Future research should focus on a more diverse population in terms of race and socioeconomic status. Implementing quantitative studies on the use of the SAFER-HOME, v. 3 assessment, in a well, older adult population would be beneficial. The descriptive qualities of this assessment were beneficial within the individual categories, but overall scores may be limited by a ceiling effect in an independent older adult population. The need for a global, yet responsive assessment in the area of home safety is important to address given the focus on health maintenance in the Affordable Care Act and a growing older adult population. A qualitative, grounded theory study focused on rapport in an online, synchronous delivery model could be beneficial across disciplines. A level of intimacy and privacy is important for health care providers and their clients, and a high level of trust is necessary for full communication. This study found that despite expertise in the topic area, greater effort was needed to develop a level of trust that may come more easily in an in-person setting. Understanding the subtleties of the online environment and how it influences rapport, client trust, and provider influence is important.

### LIMITATIONS

A limitation of this study must include the homogeneity of the participant group. Attempts were made to recruit from three different, more diverse participant pools with no responses. More intentional recruitment helped to obtain participants, but those individuals offered a less diverse perspective. Possibly because of the focus on the use of technology, individuals from lower socioeconomic backgrounds may not have had the access to the resources needed to participate. As a result, the safety issues and solutions addressed were more narrowly focused and the ability to assess the impact of technology on the process was limited.

A second limitation stems from the challenges of technology. While technology often offers solutions for many challenges, when it fails to work consistently in an educational setting, the process of reflecting on experiences, generating an understanding, and problem solving can be compromised.

With a focus on narrative learning, guided by the unique safety needs of very independent older adults, this research did not address all areas of the home, as many areas offered no past safety challenge. While the SAFER-HOME, v. 3 is a valued tool in the field of occupational therapy, the focus of this experience was educational in nature, and did not deliver typical occupational therapy services. It is possible that had the sessions been more prescriptive, the participants would have felt a greater obligation to make changes in their home, resulting in a greater change in scores. So while the SAFER-HOME, v. 3 is a reliable and valid home safety measure in occupational therapy, it may not offer statistically significant change when the intervention provided is solely education based, and delivered to a well older adult population.

A final limitation is that understanding narrative learning was clouded by occupational therapy input. For example, in addition to photos about past accidents, participants took photos of existing home modifications, and were offered concrete advice such as the proper placement of future grab bars and strategies for safe driving. These issues seemed to be a natural evolution of the occupational therapist and client discussions. Discussions about a photo did not begin with advice from the perspective of an occupational therapist (e.g., pick up your throw rugs so you don’t trip), yet when the narrative did not evolve toward an awareness of a safety problem, clients were offered specific, more prescriptive advice before the session ended. The ethical obligation to the safety of the participants was of primary importance.

### IMPLICATIONS FOR PRACTICE

Concepts from adult education can support health related fields as they are held to greater standards of accountability in practice. Client centered education is an effective way to influence positive, long term outcomes. To that end, practitioners need to have knowledge about designing effective educational experiences. It was the intention, through this multi-case study, to examine the experiences of educating older adults about home safety via telehealth using photographs as an antecedent to narrative learning. This research contributes to occupational therapy service delivery and enhances an occupational therapist’s ability to deliver home safety services via telehealth. Closer examination of how clients learn well, both within and outside of the healthcare environment, is a vital part of achieving lasting outcomes and improved quality of life for older adults.

## Figures and Tables

**Table 1:** SAFER-HOME, v. 3 Results

	Initial Visit	Final Visit	Change
**Participant 1**	14	6	−8
**Participant 2**	4	3	−1
**Participants 3 and 4**	11	8	−3
**Participant 5**	9	6	−3
**Participant 6**	4	4	0

Note. Negative scores indicate fewer home safety issues.
